# A Mechanistic Study on the Amiodarone-Induced Pulmonary Toxicity

**DOI:** 10.1155/2016/6265853

**Published:** 2016-01-10

**Authors:** Bader Al-Shammari, Mohamed Khalifa, Saleh A. Bakheet, Moustafa Yasser

**Affiliations:** ^1^Saudi Food and Drug Authority, AlKhaleej District, Prince Bander Street, P.O. Box 376067, Riyadh 11335, Saudi Arabia; ^2^Department of Pharmacology, College of Pharmacy, King Saud University, P.O. Box 2457, Riyadh 11451, Saudi Arabia

## Abstract

Amiodarone- (AM-) induced pulmonary toxicity (AIPT) is still a matter of research and is poorly understood. In attempting to resolve this issue, we treated Sprague-Dawley rats with AM doses of 80 mg/kg/day/i.p. for one, two, three, and four weeks. The rats were weighed at days 7, 14, 21, and 28 and bronchoalveolar lavages (BAL) were obtained to determine total leukocyte count (TLC). For each group, lung weighing, histopathology, and homogenization were performed. Fresh homogenates were used for determination of ATP content, lipid peroxides, GSH, catalase, SOD, GPx, GR activities, NO, and hydroxyproline levels. The results showed a significant decrease in body weight and GSH depletion together with an increase in both lung weight and lung/body weight coefficient in the first week. Considerable increases in lung hydroxyproline level with some histopathological alterations were apparent. Treatment for two weeks produced a significant increase in BAL fluid, TLC, GR activity, and NO level in lung homogenate. The loss of cellular ATP and inhibition of most antioxidative protective enzymatic system appeared along with alteration in SOD activity following daily treatment for three weeks, while, in rats treated with AM for four weeks, more severe toxicity was apparent. Histopathological diagnosis was mostly granulomatous inflammation and interstitial pneumonitis in rats treated for three and four weeks, respectively. As shown, it is obvious that slow oedema formation is the only initiating factor of AIPT; all other mechanisms may occur as a consequence.

## 1. Introduction

Amiodarone (AM) is benzofuran derivative with highly effective class III antidysrhythmic activity. It is used for treating ventricular and supraventricular dysrhythmia and may have a role in postmyocardial infraction mortality reduction [1]. However, it is associated with many side effects involving many different organ systems [2]. The most serious side effect of amiodarone is pulmonary toxicity. Amiodarone-induced pulmonary toxicity (AIPT) is characterized in part by oedema, phospholipidosis, inflammation and thickening of the alveolar septa, intra-alveolar inflammation, and pulmonary fibrosis [3–5]. The etiology of AIPT is unknown. However, several causes have been proposed, including direct or indirect toxicity. It has also been postulated that the cause is complex and multifactorial, possibly involving several mechanisms [6].

Consequently, the mechanism of the adverse reaction is still a matter of research. Thus, the overall objective of this study was to shed light on the possible mechanism(s) associated with AIPT in rats.

## 2. Materials and Methods

### 2.1. Drug

Amiodarone was obtained from MP Biomedical, USA. Amiodarone solution was freshly prepared by dissolving it in distilled water at 65°C and allowing it to cool to room temperature before use in the experiments [7–9].

### 2.2. Chemicals

Chemicals were obtained from the suppliers as follows. Calcium-free and magnesium-free phosphate-buffered saline (PBS) was obtained from MP Biomedicals, Santa Ana, CA, USA. Thiobarbituric acid (TBA) was obtained from Fluka, Buchs, Switzerland. Ellman's reagent (5.5′-dithiobis-(2-nitrobenzoic acid)) (DTNB), sulfanilamide, adenosine triphosphate (ATP), sodium nitrate, and the chemical for measurement of glutathione reductase (GR) activity were obtained from Sigma-Aldrich, St. Louis, MO, USA. Chloramine-T and sodium dodecyl sulfate (SDS) were obtained from Winlab Laboratory Chemicals, Leicestershire, UK. N-(Naphthyl)-ethylenediamine dihydrochloride (NEDD) was obtained from Riedel-de Haёn, D3010 Seelze, Germany. 4-Dimethylaminobenzaldehyde was obtained from BDH Chemicals Ltd., Poole, UK. Diagnostic kits for measurement of superoxide dismutase (SOD) and glutathione peroxidase (GPx) were obtained from Randox Laboratories Ltd., Crumlin, UK. All other chemicals were of highest analytical grade.

### 2.3. Animals and Treatments

Adult Sprague-Dawley rats (Experimental Animal Care Centre, College of Pharmacy, KSU, SA), weighing 130–150 g, were maintained on a 12 h light/12 h dark cycle and fed a standard animal pellet diet with free access to water.

Rats were randomly allocated into eight groups (10 animals/group). Amiodarone (or equivalent volume of vehicle for control groups) was given 80 mg/kg/day/i.p. for 1, 2, 3, and 4 weeks for treated groups. Rats underwent the experiment protocols at days 7, 14, 21, and 28 following amiodarone injections of their respective group. The dose of amiodarone used in the present study was selected based on the previous report of Kannan et al. [10].

### 2.4. Total Leukocyte Count in Bronchoalveolar Lavage Fluid BALF

Rats were anesthetized using a mixture of ketamine (90 mg/kg/i.p.) and xylazine (10 mg/kg/i.p.). An incision was made in the trachea and 5 mL of PBS at 37°C was slowly injected into the trachea by catheter needle. This was repeated two more times so that approximately 15 mL was recovered and kept on ice. Lavage samples were used immediately to measure total leukocyte counts [11].

Total leukocyte counts were measured according to the method of Barbara and Stanley [12]. In brief, cell viability of lung cell was measured using the trypan blue dye exclusion technique. Equal volumes of lung cell suspension and trypan blue stain (0.047%) were mixed and a drop on the hemocytometer. The viable cells (unstained) were counted with a low power light microscope.

### 2.5. Isolation and Homogenization of Lung Samples

After withdrawal of BALF, the lungs were excised and immersed into saline, blotted, weighed, placed directly in liquid nitrogen, and stored at −80°C for further analysis. Homogenization was done freshly. The homogenate was used for determination of biochemical parameters and the freshly removed lungs fragments were fixed in 10% formalin for histopathology.

### 2.6. Calculation of Lung/Body Weight Coefficient

Total body weight, lung wet weight, and relative lung/body coefficient were calculated. The lung/body coefficient was calculated as previously reported by Chen et al. [13].

### 2.7. Determination of Lipid Peroxides (MDA) and Reduced Glutathione (GSH) Content in Lung Homogenates

The lipid peroxide level in lung homogenate was determined as thiobarbituric acid-reactive substances spectrophotometrically at an absorbance of 532 nm, by the method of Ohkawa et al. [14], and the concentrations were expressed as nmole malondialdehyde (MDA) per gram tissue. Tissue levels of the acid soluble thiols, mainly reduced glutathione GSH, were assayed spectrophotometrically at 412 nm, according to the method of Ellman [15]. The GSH content was expressed as *μ*mol/g lung.

### 2.8. Determination of Nitric Oxide (NO_*x*_) in Lung Homogenates

The tissue level of total nitrate/nitrite (NO_*x*_) was determined by the acidic Griess reaction. Prior to the Griess reaction all nitrate was converted to nitrite using vanadium trichloride as described by Miranda et al. [16].

### 2.9. Determination of Antioxidant Enzymes in Lung Homogenates

Glutathione reductase was measured by monitoring the oxidation of NADPH at 340 nm in presence of oxidized glutathione. The glutathione reductase activity is expressed as nmol/min/g lung according to the glutathione reductase assay kit (Sigma-Aldrich, St. Louis, MO, USA) instructions.

Glutathione peroxidase was measured by monitoring the oxidation of NADPH at 340 nm according to the method of Paglia and Valentine [17]. The glutathione peroxidase activity is expressed as nmol/min/g lung.

Catalase (CAT) activity was determined spectrophotometrically according to the method of Higgins et al. [18], namely, via the assay of hydrogen peroxide (H_2_O_2_). Catalase activity is expressed as *μ*mol/min/g lung using a molar absorbance of 43.6 for H_2_O_2_.

The superoxide dismutase assay was a slight modification of the indirect inhibition assay developed by McCord and Fridovich [19]. Superoxide dismutase was measured by monitoring the decrease of the rate of detector 2(4-iodophenyl)-3-(4-nitrophenol)-5-phenyltetrazolium chloride (INT) reaction with superoxide anion. The superoxide dismutase activity was measured at 505 nm and is expressed as U/g tissue.

### 2.10. Determination of Adenosine Triphosphate in Lung Homogenates

Adenosine triphosphate (ATP) levels were determined in lung homogenates using HPLC according to the method reported by Botker et al. [20]. In brief, kidney tissues were homogenized in ice-cold 6% perchloric acid and centrifuged at 110 g for 15 min at 0.5°C and the supernatant was injected onto the HPLC after neutralization to pH 6-7. Chromatographic separation was performed at a flow rate of 1.2 mL/min, using ODS-Hypersil, 150 × 4.6 mm I.D. 5 mm column (Supelco S.A., Gland, Switzerland) and 75 mmol/L ammonium dihydrogen phosphate as the mobile phase. The peak elution was followed at 254 nm.

### 2.11. Determination of Hydroxyproline in Lung Homogenates

Hydroxyproline was determined as a biochemical index of fibrosis. In brief, 10 *μ*L of lung homogenates was hydrolyzed in 40 *μ*L of 2 N NaOH at 120°C for 20 min. Absorbance of each sample was read at 550 nm and the concentration of hydroxyproline was determined from the standard curve as *μ*g/g lung according to Reddy and Enwemeka [21].

### 2.12. Histopathological Examination

Lung specimens were fixed in formalin (10%) for 24 hrs, until the tissue became hard enough to be sectioned. Tissues were then embedded in paraffin wax, serially sectioned (5 *μ*m in thickness), and stained with hematoxylin for 10 min then counterstained in eosin for 1 min, followed by rapid rinsing in distilled water. Finally, tissues were dehydrated, mounted, and examined using a light microscope (United Medical Laboratories, Riyadh, Saudi Arabia).

### 2.13. Statistical Analysis

Data are expressed as mean ± standard error (SE). Statistical comparison between treatment and control groups in each week was done using Student's *t*-test. One-way analysis of variance (ANOVA) followed by Tukey-Kramer multiple comparisons test and Wilcoxon's signed rank test for statistical comparison between more than two groups measures using a software computer program (Graphpad InStat, Version 3). Significance was accepted at *P* < 0.05.

## 3. Results

**Figure 1:**
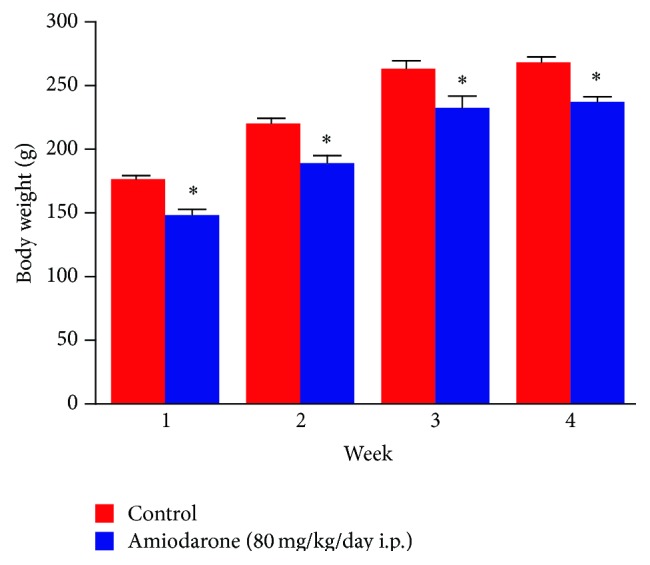
The effect of amiodarone on rat body weight. Amiodarone was given (80 mg/kg/day i.p.) for one, two, three, and four weeks. Each group was compared with its respective control. The body weight was determined on days 7, 14, 21, and 28. The data represent the mean ± SEM of 10 rats. ^*∗*^Significant difference from control group (*P* < 0.05).

**Figure 2:**
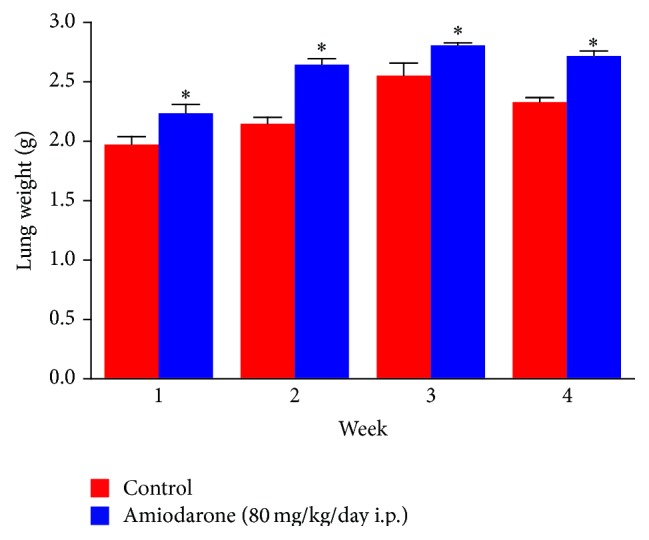
The effect of amiodarone on rat lung weight. Amiodarone was given (80 mg/kg/day i.p.) for one, two, three, and four weeks. Each group was compared with its respective control. The lung weight was determined on days 7, 14, 21, and 28. The data represent the mean ± SEM of 10 rats. ^*∗*^Significant difference from control group (*P* < 0.05).

**Figure 3:**
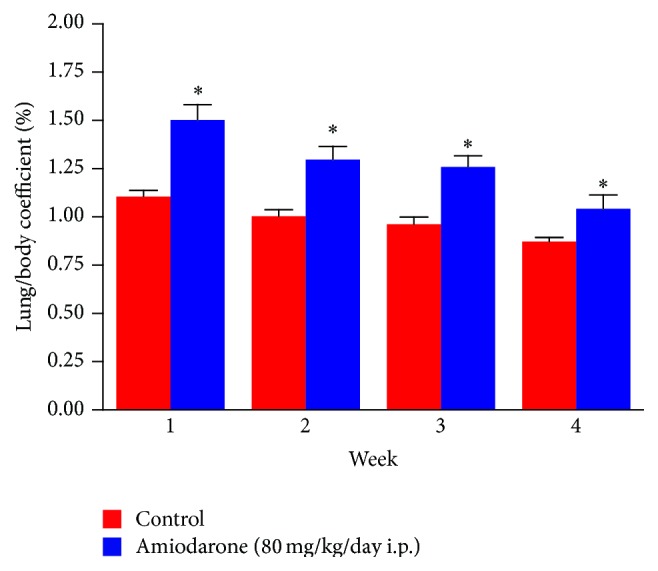
The effect of amiodarone on rat lung/body coefficient. Amiodarone was given (80 mg/kg/day i.p.) for one, two, three, and four weeks. Each group was compared with its respective control. The lung/body coefficient was calculated on days 7, 14, 21, and 28. The data represent the mean ± SEM of 10 rats. ^*∗*^Significant difference from control group (*P* < 0.05).

**Figure 4:**
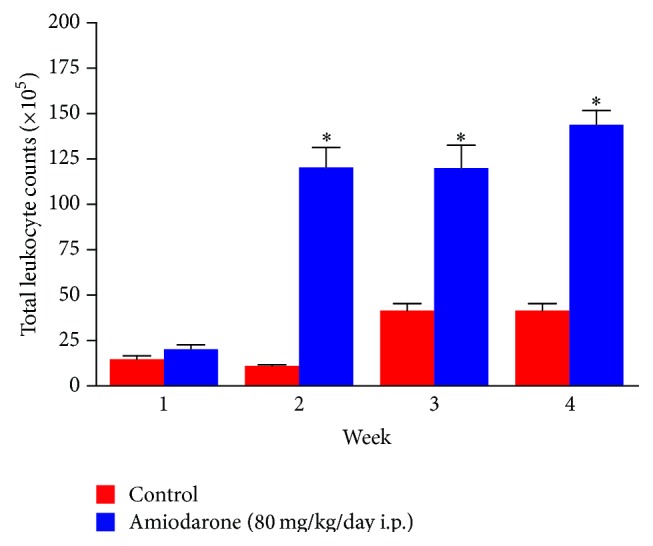
The effect of amiodarone on total leukocyte count in bronchoalveolar lavage fluid of rat. Amiodarone was given (80 mg/kg/day i.p.) for one, two, three, and four weeks. Each group was compared with its respective control. The total leukocyte count was calculated on days 7, 14, 21, and 28. The data represent the mean ± SEM of 10 rats. ^*∗*^Significant difference from control group (*P* < 0.01).

The results of the study revealed that daily intraperitoneal administration of AM (80 mg/kg/day i.p.) for one, two, three, and four weeks resulted in a significant decrease in body weight (Figure 1), indicating that the animals had reacted adversely to the amiodarone treatment. The significant increase in wet lung weight (Figure 2) and lung/body coefficient (Figure 3) and the large increase in total cell count number in BALF (Figure 4) indicated that oedema started early and was followed by inflammation.

**Figure 5:**
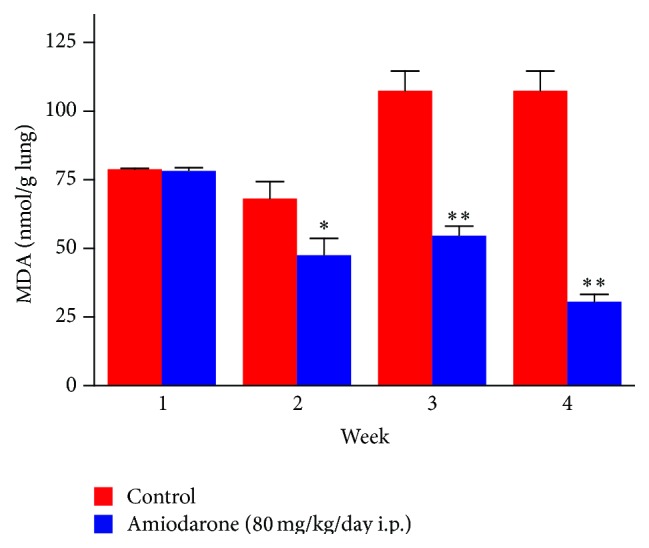
The effect of amiodarone on malondialdehyde MDA level in lung rat homogenate. Amiodarone was given (80 mg/kg/day i.p.) for one, two, three, and four weeks. MDA was measured on days 7, 14, 21, and 28. The data represent the mean ± SEM of 10 rats. ^*∗*^Significant difference from control group (*P* < 0.05). ^*∗∗*^Significant difference from control group (*P* < 0.01).

**Figure 6:**
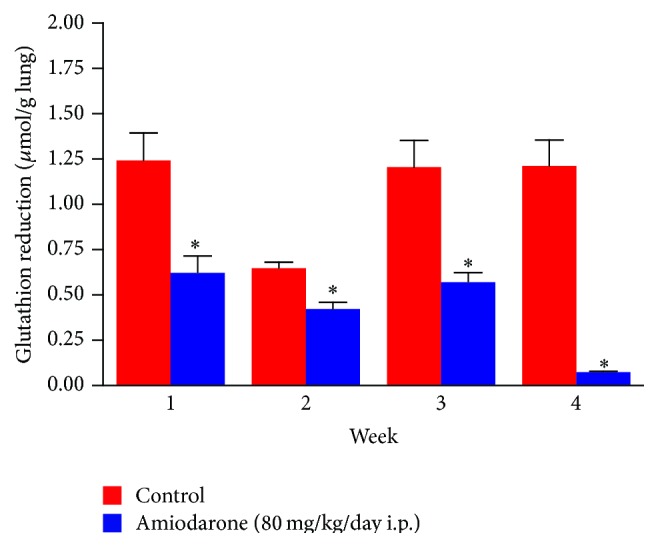
The effect of amiodarone on reduced glutathione (GSH) level in rat lung homogenate. Amiodarone was given (80 mg/kg/day i.p.) for one, two, three, and four weeks. Each group was compared with its respective control. The reduced glutathione was measured on days 7, 14, 21, and 28. The data represent the mean ± SEM of 10 rats. ^*∗*^Significant difference from control group (*P* < 0.01).

AM given to rats in a dose of 80 mg/kg/day i.p. decreased the lung content of malondialdehyde (MDA) (Figure 5) compared to the value obtained from the control group (68.10 ± 6.27 to 47.58 ± 6.10, 107.43 ± 7.10 to 45.61 ± 3.52, and 107.4 ± 7.1 to 30.56 ± 2.64 nmol/g lung for two, three, and four weeks, resp.), which can be explained as indicated as part of the antioxidant effect of AM. Throughout all the treatment periods, AM decreased the GSH level significantly in lung homogenate (Figure 6). The level was decreased by 50% after one week, 34.8% after two weeks, 52.6% after three weeks, and ~100% after four weeks as compared with the untreated group following intraperitoneal administration of AM.

**Figure 7:**
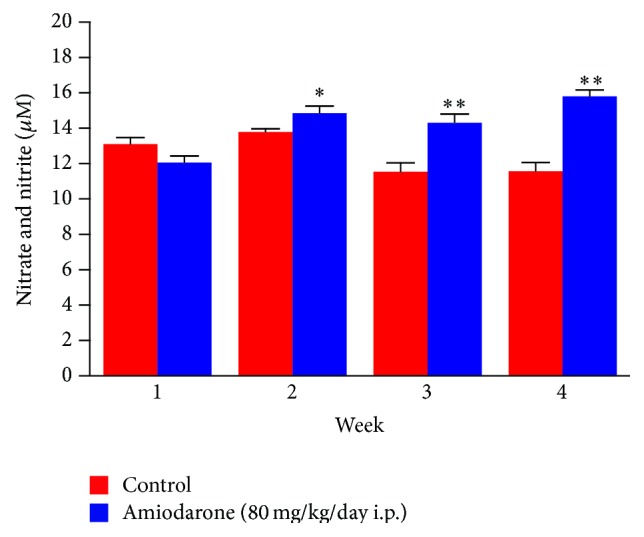
The effect of amiodarone on nitric oxide as nitrate/nitrite concentration in rat lung homogenate. Amiodarone was given (80 mg/kg/day i.p.) for one, two, three, and four weeks. Each group was compared with its respective control. The nitrate and nitrite concentration was measured on days 7, 14, 21, and 28. The data represent the mean ± SEM of 10 rats. ^*∗*^Significant difference from control group (*P* < 0.05). ^*∗∗*^Significant difference from control group (*P* < 0.01).

**Figure 8:**
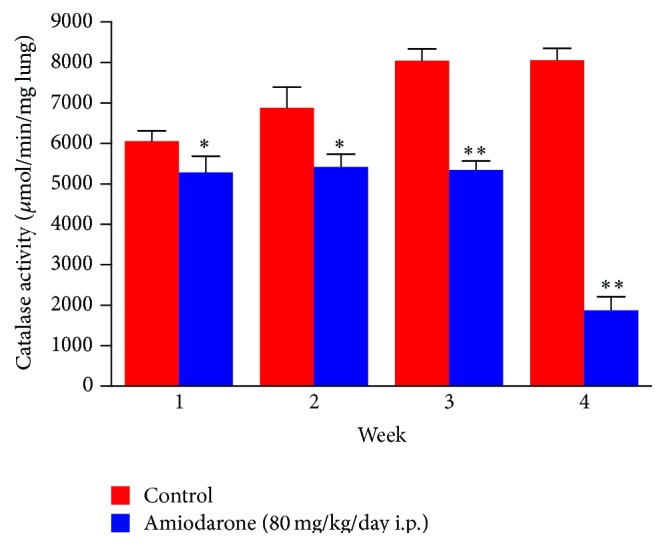
The effect of amiodarone on catalase activity in rat lung homogenate. Amiodarone was given (80 mg/kg/day i.p.) for one, two, three, and four weeks. Each group was compared with its respective control. The catalase activity was measured on days 7, 14, 21, and 28. The data represent the mean ± SEM of 10 rats. ^*∗*^Significant difference from control group (*P* < 0.05). ^*∗∗*^Significant difference from control group (*P* < 0.01).

**Figure 9:**
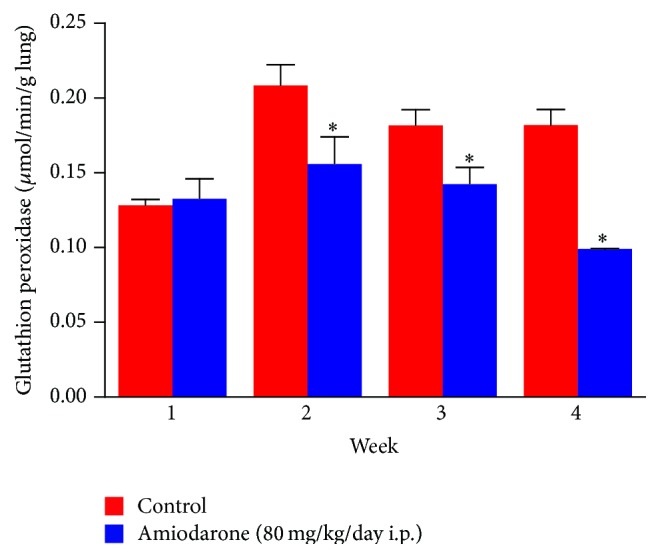
The effect of amiodarone on glutathione peroxidase activity in rat lung homogenate. Amiodarone was given (80 mg/kg/day i.p.) for one, two, three, and four weeks. Each group was compared with its respective control. The glutathione peroxidase activity was measured on days 7, 14, 21, and 28. The data represent the mean ± SEM of 10 rats. ^*∗*^Significant difference from control group (*P* < 0.05).

**Figure 10:**
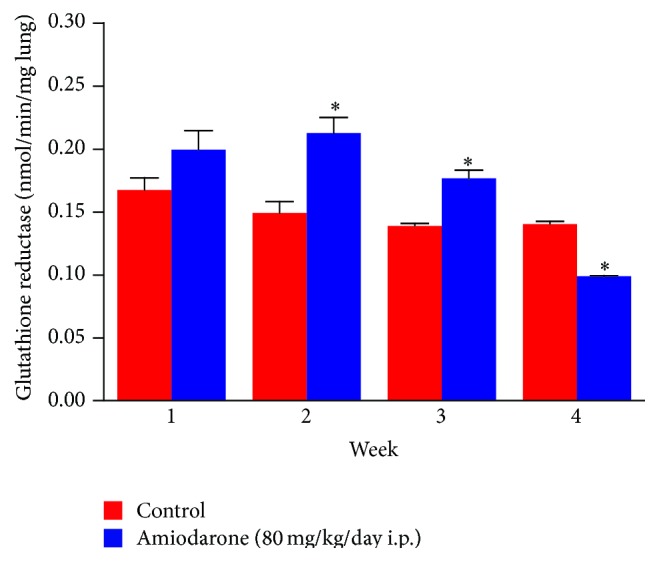
The effect of amiodarone on glutathione reductase activity in rat lung homogenate. Amiodarone was given (80 mg/kg/day i.p.) for one, two, three, and four weeks. Each group was compared with its respective group. The glutathione reductase activity was measured on days 7, 14, 21, and 28. The data represent the mean ± SEM of 10 rats. ^*∗*^Significant difference from control group (*P* < 0.05).

**Figure 11:**
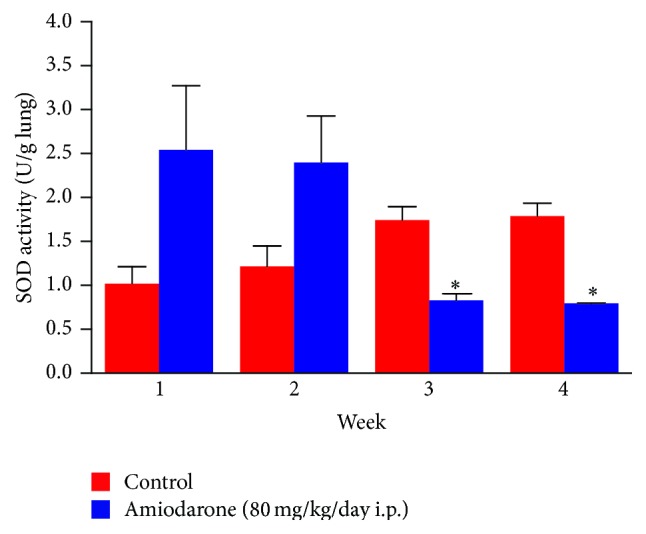
The effect of amiodarone on superoxide dismutase activity in rat lung homogenate. Amiodarone was given (80 mg/kg/day i.p.) for one, two, three, and four weeks. Each group was compared with its respective control. The superoxide dismutase activity was measured on days 7, 14, 21, and 28. The data represents the mean ± SEM of 10 rats. ^*∗*^Significant difference from control group (*P* < 0.01).

In the current study, daily administration of amiodarone for two, three, and four weeks resulted in a significant increase in nitrate/nitrite concentration in the lung homogenate compared to a control value (Figure 7). However, administration of AM for two weeks resulted in a significant decrease in the level of catalase (Figure 8) and glutathione peroxidase (Figure 9) together with a significant increase in glutathione reductase activity (Figure 10). The same results were obtained following daily administration of amiodarone for three weeks; the only difference was the observed decrease in the SOD level (Figure 11). Daily administration of amiodarone for four weeks produced a significant decrease in the activity of all antioxidant enzymes.

**Figure 12:**
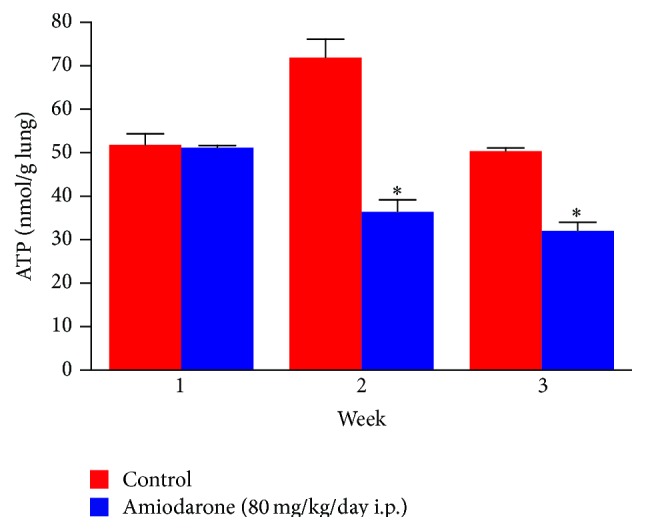
The effect of amiodarone on ATP level in rat lung homogenate. Amiodarone was given (80 mg/kg/day i.p.) for one, two, and three weeks. The ATP level was measured on days 7, 14, and 21. The data represent the mean ± SEM of 10 rats. ^*∗*^Significant difference from control group (*P* < 0.01).

The results obtained in the current study clearly demonstrate that daily administration of amiodarone for two and three weeks produced a significant depletion in ATP level (Figure 12) from 71.88 ± 4.25 to 36.45 ± 2.69 nmol/g lung and from 50.37 ± 0.73 to 32.05 ± 2.01 nmol/g lung, respectively.

**Figure 13:**
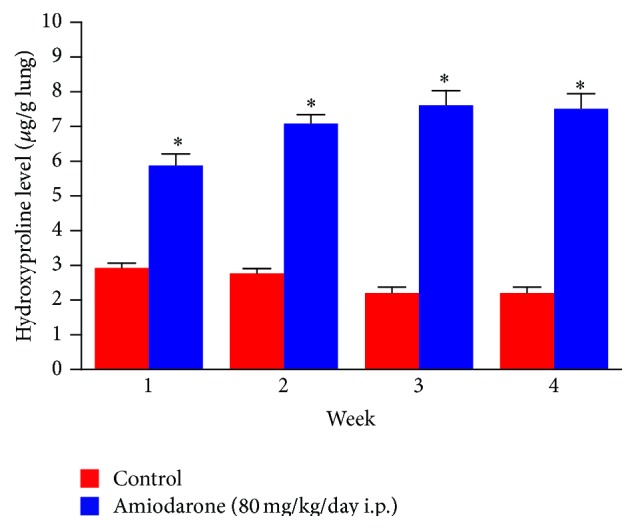
The effect of amiodarone on hydroxyproline content of rat lung homogenate. Amiodarone was given (80 mg/kg/day i.p.) for one, two, three, and four weeks. Each group was compared with its respective control. The hydroxyproline content was measured on days 7, 14, 21, and 28. The data represents the mean ± SEM of 10 rats. ^*∗*^Significant difference from control group (*P* < 0.01).

**Figure 14:**
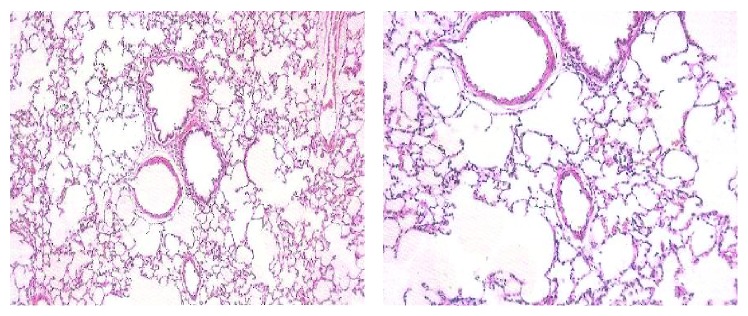
Histopathology of control rat. Microscopic description: normal rat lung.

**Figure 15 fig15:**
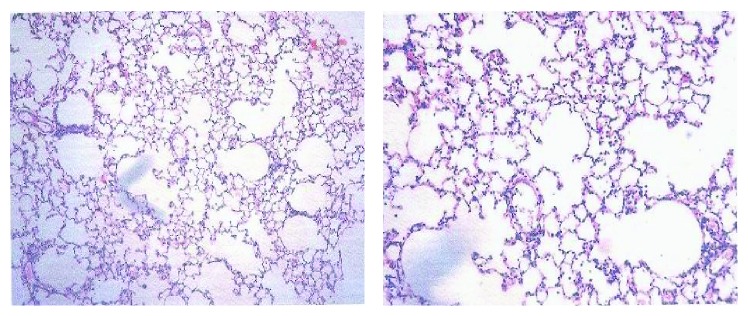
Effect of amiodarone on rat lung histopathology. Amiodarone was given (80 mg/kg/day i.p.) for one week. Microscopic description: examination reveals fragments of lung tissue showing vascular congestion, unremarkable bronchi, and interstitial capillary dilation with some lymphocytes.

**Figure 16 fig16:**
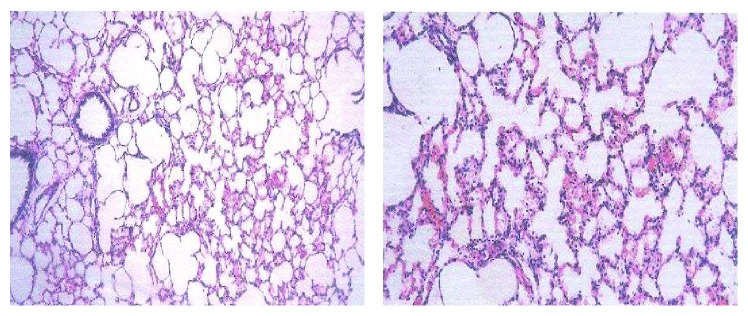
Effect of amiodarone on rat lung histopathology. Amiodarone was given (80 mg/kg/day i.p.) for two weeks. Microscopic description: examination reveals fragments of lung tissue showing interstitial vascular congestion and moderate lymphocytic infiltrate. The bronchi are unremarkable.

**Figure 17 fig17:**
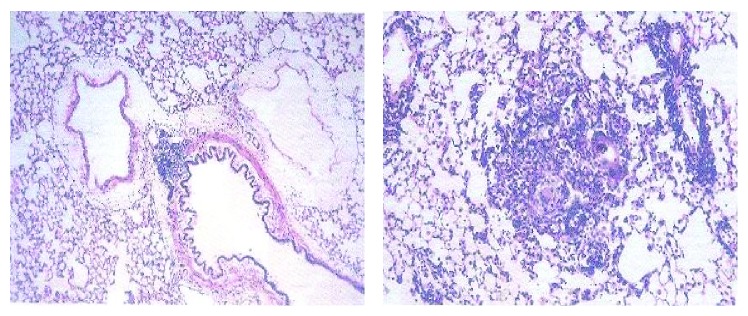
Effect of amiodarone on rat lung histopathology. Amiodarone was given (80 mg/kg/day i.p.) for three weeks. Microscopic description: inflamed lung tissue showing nodular collection of lymphocytes and subpleural granulomas with multinucleated giant cells. The lung tissue is emphysematous and congested. Histopathologic diagnosis: granulomatous inflammation.

**Figure 18:**
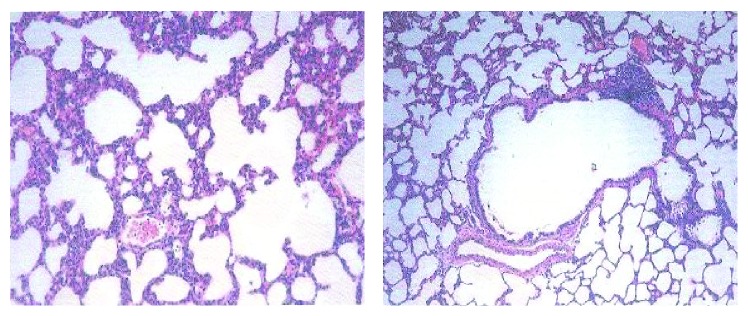
Effect of amiodarone on rat lung histopathology. Amiodarone was given (80 mg/kg/day i.p.) for four weeks. Microscopic description: emphysematous lung tissue showing nodular lymphocytic collection and thickened alveolar walls that contain lymphocytes and neutrophils. Histopathologic diagnosis: interstitial pneumonitis.

The lung hydroxyproline of amiodarone-treated groups was significantly increased (Figure 13) by approximately 50%, 61%, 71%, and 70.6% at one, two, three, and four weeks, respectively, as compared to the water control groups. Histopathological diagnosis was mostly interstitial capillary dilation with some lymphocytes, granulomatous inflammation, interstitial pneumonitis, and thickened alveolar walls in the treated groups (Figures 14 [Fig fig15] [Fig fig16] [Fig fig17]–18).

## 4. Discussion

It is evident that the most important characteristic features obtained following daily i.p. administration of amiodarone in rats for one week include decreased body weight together with an increase in both lung weight and lung/body weight coefficient. Glutathione depletion was observed. Considerable increases in lung hydroxyproline level with some histopathological alterations are apparent.

The observed change in lung glutathione status is, therefore, indicative of specific pulmonary response to amiodarone exposure, which is in good agreement with data obtained from several investigations.

Further studies have revealed that amiodarone is metabolized to an aryl radical that may give rise to other reactive oxygen species [22, 23].

Both catalytic and scavenger antioxidants have been shown to attenuate amiodarone-induced lung injury and fibrosis in animals [24].

Reactive oxygen species (ROS, e.g., H_2_O_2_) cause endothelial injury leading to oedema, thrombosis, and inflammation, contributing to morbidity and mortality in acute lung injury (ALI), ischemia-reperfusion (I/R), and many other disease conditions [25–30].

ROS cause endothelial dysfunction manifested by increased permeability, leukocyte recruitment, adhesion and transmigration, thrombosis, and other pathways initiating and propagating inflammation [31, 32]. However, current means for vascular protection have provided inconsistent results in many animal and clinical studies, at least in part due to suboptimal delivery of antioxidants to the endothelial cells.

On the other hand, the pulmonary endothelium represents the source of ROS generated via diverse enzymatic mechanisms by leukocytes, alveolar macrophages, and endothelial cells themselves [31, 33–35].

Indicators of general lung cell injury, including lung weights and lung/body weight coefficient, all increased early after amiodarone dosing and remained elevated at every time period examined. Interstitial capillary dilatation with some lymphocytes produced by amiodarone in the present study may explain the effect of the drug in increasing capillary permeability and subsequent oedema formation. The increased hydroxyproline level may correlate with oedema that occurs early after amiodarone administration and infiltration by inflammatory cells days after.

The data obtained in the present study suggest that inflammation is absent in early AIPT and may occur as a consequence of lung oedema or the process that leads to oedema formation (Figures 1–4).

In rats treated with amiodarone for two weeks, an increased total leukocyte count is observed in BALF. Such increase is an informative measure of inflammation in the lung.

The elevation of glutathione reductase activity observed most likely represents inflammation of lung and the associated influx of glutathione reductase-containing inflammatory cells rather than an adaptive induction due to oxidative stress. Loss of cellular ATP can be considered as a pivotal event in the initiation of amiodarone cytotoxicity in the lung. The inhibition of most of the antioxidative protective enzymatic system supports the generation of more ROS and subsequent role of oxidative stress in AIPT.

The alteration in SOD activity produced by administration of amiodarone for three weeks is indicative of changes in the handling of ROS.

The increased NO observed is indicative of oxidant-related tissue injury by formation of highly reactive nitrogen intermediates. For example, NO reacts with superoxide and generates a highly reactive metabolite, peroxynitrite (OONO^−^), which is presumed to be largely responsible for most of the adverse effects of excessive generation of NO [36]. Since this reaction occurs at a nearly diffusion limited rate, it is assumed that NO can outcompete superoxide dismutases (SOD) for reaction with O_2_^−^ and that OONO^−^ will be generated as a consequence of the simultaneous production of O_2_^−^ and NO [37]. Thus, the role of NO in AIPT cannot be ruled out. Targeting SOD or SOD mimetics may help to decompose superoxide anion and thus prevent inactivation of nitric oxide and oxidative nitration in the tissues [38, 39].

The study was extended to a fourth week in a trial to follow up the progression of AIPT; more severe toxicity is apparent with approximately the same changes seen in rats treated with amiodarone for three weeks except for a significant reduction in glutathione reductase activity which can be explained as a sort of imbalance between oxidant and antioxidant systems. Daily i.p. administrations of amiodarone for five weeks resulted in a considerable mortality.

In conclusion, it is evident that AIPT occurs in rats following daily i.p. administration in a sequence of oedema formation, inflammatory reactions, and severe imbalance between ROS production and antioxidant defenses (oxidative stress) ending ultimately in severe lung toxicity. Such oedema may lead to pulmonary fibrosis and we think the future research should focus on better understanding of the mechanisms involved in oedema formation by amiodarone.
